# Structure-Controllable Synthesis of Multiferroic YFeO_3_ Nanopowders and Their Optical and Magnetic Properties

**DOI:** 10.3390/ma10060626

**Published:** 2017-06-07

**Authors:** Meng Wang, Ting Wang, Shenhua Song, Manlin Tan

**Affiliations:** 1 Shenzhen Key Laboratory of Advanced Materials, Department of Materials Science and Engineering, Shenzhen Graduate School, Harbin Institute of Technology, Shenzhen 518055, China; wangmeng1985@hit.edu.cn (M.W.); twang-hawk@foxmail.com (T.W.); 2 Research Institute of Tsinghua University in Shenzhen, Shenzhen 518055, China

**Keywords:** hexagonal YFeO_3_, orthorhombic YFeO_3_, optical materials and properties, magnetic materials

## Abstract

Phase-pure hexagonal and orthorhombic YFeO_3_ nanopowders are synthesized by low-temperature solid-state reaction along with Zr doping. The obtained powders are characterized by X-ray diffraction, field emission scanning electron microscopy, and physical property measurements. The hexagonal YFeO_3_ exhibits a narrower optical band gap in comparison to the orthorhombic one, while the orthorhombic YFeO_3_ presents better magnetic properties. The formation of hexagonal or orthorhombic phase can be effectively controlled by Zr doping. The temperature range of synthesizing the hexagonal YFeO_3_ nanopowders is increased by ~200 °C due to Zr doping so that they can be easily synthesized, which possesses a finer particle size and a smaller optical band gap, making it favorable for optical applications.

## 1. Introduction

As a typical one of the second generation multiferroic materials, YFeO_3_, with its low Curie temperature (~−256 °C) and high Neel temperature (~370 °C), can couple both ferroelectric and anti-ferromagnetic orderings [1,2,3,4,5,6]. It is known that orthorhombic YFeO_3_ possesses significant magnetic properties, while the hexagonal YFeO_3_ owns better photocatalytic properties and a smaller optical band gap (1.96 eV) [7]. However, to synthesize a stable hexagonal YFeO_3_ is difficult. As the calcination temperature increases, the hexagonal phase is prone to transform to the orthorhombic phase. Previous studies show that the hexagonal phase can be obtained only in the range of 700–720 °C with the sol-gel method, while orthorhombic YFeO_3_ and other minors (Y_2_O_3_ or Fe_2_O_3_) co-exist [7,8,9]. The calcination temperature gap is so narrow that no pure hexagonal phase can be obtained.

Chemical doping is an effective way to improve the material properties and has been successfully used in YFeO_3_, including Mn and Cr doping at the Fe site [10,11] and Gd doping at Y site [12]. All the studies are related to the magnetic enhancement and not associated with the optical properties. Since Zr ions have a larger radius and a higher valence compared with Fe ions, Zr-doping can lead to a change of the crystal lattice, which may affect the magnetic and optical properties. In the present work, Zr doping at the Fe site is employed to synthesize YFeO_3_ nanopowders with a controlled phase structure. The effect of Zr doping and calcination temperature on phase structure is studied, and the optical and magnetic properties of YFeO_3_ with various phase compositions are investigated.

## 2. Experimental Procedures

YFeO_3_, YFe_0.95_Zr_0.05_O_3_, and YFe_0.90_Zr_0.10_O_3_ nanopowders were synthesized by a low-temperature solid-state reaction. The reagents were Y(NO_3_)_3_·6H_2_O (Aladdin, >99%), Fe(NO_3_)_3_·9H_2_O (Aladdin, >99%), Zr(CH_3_COO)_4_ (Aladdin, >15–16%), and citric acid (Aladdin, >99.5%). The method of precursor preparation was already given in [13]. The precursor was grounded into powders and subsequently calcined for 1 h in air at 700, 750, and 800 °C to obtain pure YFeO_3_ nanopowders (denoted as p-700, p-750, and p-800, respectively), and calcined at 800, 900, and 950 °C to obtain YFe_0.95_Zr_0.05_O_3_ and YFe_0.90_Zr_0.10_O_3_ nanopowders (denoted as z5-800, z5-900 and z5-950; z10-800, z10-900 and z10-950, respectively). The crystal structures of the powders were analysed using X-ray Diffraction (XRD, D/max-RB, Rigaku, Japan). The morphologies of the nanopowders were observed using scanning electron microscopy equipped with energy dispersive X-ray spectroscopy (S-4700, Hitachi, Tokyo, Japan). Magnetic measurements were conducted at room temperature with a physical property measurement system (PPMS, QUANTUM DESIGN, DynaCool-9T, San Diego, CA, USA). Absorbance spectra of YFeO_3_ powders were obtained using UV–visible spectroscopy (SHIMADZU, UV-2600, Kyoto, Japan).

## 3. Results and Discussion

EDS analysis of the Zr-doped YFeO_3_ powders is conducted. The atomic ratios of Y:Zr:Fe for z5-800, z5-900, and z5-950 are 1:0.049:0.949, 1:0.051:0.951, and 1:0.048:0.954, respectively, which are close to 1:0.05:0.95. Similarly, the atomic ratios of Y:Zr:Fe of z10-800, z10-900, and z10-950 are 1:0.098:0.901, 1:0.101:0.900, and 1:0.096:0.903, respectively, which are close to 1:0.10:0.90. Therefore, the stoichiometry is maintained in the calcined powders.

**Figure 1:**
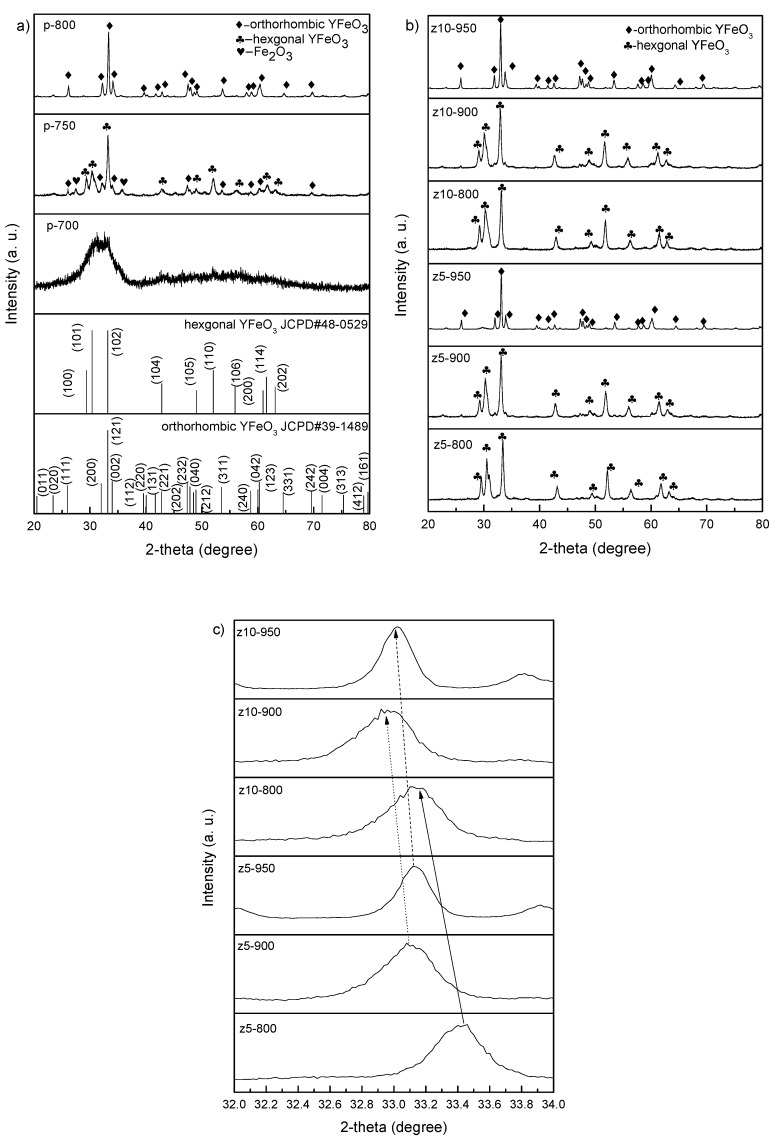
XRD patterns of (**a**) pure YFeO_3_; (**b**) YFe_0.95_Zr_0.05_O_3_ and YFe_0.90_Zr_0.10_O_3_ synthesized at different calcination temperatures and (**c**) the magnified patterns at 2θ ~ 33°.

Figure 1a represents the XRD patterns of the undoped YFeO_3_ synthesized at several calcination temperatures. As can be seen, the crystalline YFeO_3_ cannot be synthesized at 700 °C because the calcination temperature is too low and the mixed hexagonal and orthorhombic YFeO_3_ can be merely obtained at 750 °C. At 800 °C, the pure orthorhombic YFeO_3_ is synthesized, implying that the phase-pure hexagonal YFeO_3_ cannot be synthesized at any calcination temperature. Figure 1b shows the XRD patterns of the YFe_0.95_Zr_0.05_O_3_ and YFe_0.90_Zr_0.10_O_3_ synthesized at different calcination temperatures. z5-800 and z5-900 exhibit sharp and apparent hexagonal peaks without any visible impurities, while z5-950 shows sharp and apparent orthorhombic peaks. For YFe_0.90_Zr_0.10_O_3_, the XRD patterns are similar to those for YFe_0.95_Zr_0.05_O_3_. Figure 1c shows the magnified patterns of peaks at 2θ ~ 33°. The peaks of the samples calcined at all temperatures shift to lower 2θ angles with increasing Zr content, which arises from the larger radius of Zr ion (Zr^4+^ 0.73 Å) compared to Fe ion (0.645 Å for Fe^3+^). This means that the Zr ions partially occupy the Fe sites in YFeO_3_. Consequently, it is also concluded that the hexagonal phase can be well synthesized between 800 and 900 °C. The hexagonal phase is thermodynamically stable below ~740 °C for pure YFeO_3_. When the calcination temperature reaches 740–780 °C, the hexagonal YFeO_3_ will transform to the orthorhombic YFeO_3_, so that it is difficult to synthesize pure hexagonal phase YFeO_3_ in practice [14,15]. However, this transition temperature may be raised by ~200 °C due to Zr doping, so that the perfect hexagonal phase can be synthesized between 800 and 900 °C with the aid of Zr doping, but it cannot be for the undoped YFeO_3_.

Compared with the orthorhombic YFeO_3_, the hexagonal YFeO_3_ is metastable. The stability of the orthorhombic YFeO_3_ phase can be evaluated by the tolerance factor (*t*) [16]:

(1) $$t=\frac{R_{Y}+R_{O}}{\sqrt{2}\left(R_{\mathrm{Fe}}+R_{O}\right)}$$

*R*_Y_, *R*_O_, and *R*_Fe_ represent the ionic radii of Y, O, and Fe ions, respectively. Normally, the tolerance factor for the stable orthorhombic phase is in the range of 0.8 and 1 [17]. With the doping of Zr, *t* changes from 0.79 to 0.75, and thus it becomes more unstable, while the hexagonal phase becomes more stable. Furthermore, the high valence of Zr can lead to lattice distortion, thereby resulting in instability of orthorhombic phase. Therefore, the hexagonal phase is prone to formation. Nevertheless, since the orthorhombic phase is stable at higher temperatures, the hexagonal phase will finally transform to the orthorhombic phase when the calcination temperature increases to a certain temperature.

**Figure 2:**
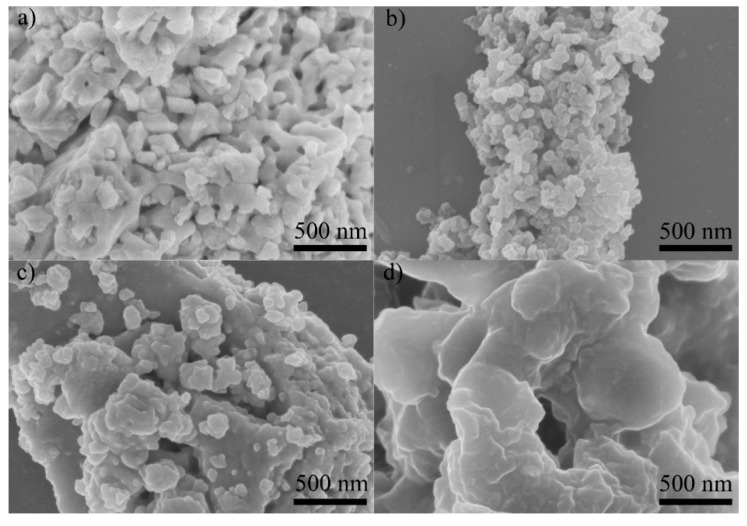
SEM micrographs for (**a**) p-800; (**b**) z5-800; (**c**) z5-900 and (**d**) z5-950 samples.

Since the XRD patterns of YFe_0.95_Zr_0.05_O_3_ and YFe_0.90_Zr_0.10_O_3_ are similar to each other, the morphology, optical, and magnetic properties of YFe_0.95_Zr_0.05_O_3_ nanopowders are only discussed. Figure 2 shows the SEM micrographs of p-800, z5-800, z5-900, and z5-950. The particles of p-800 appear to be homogeneous in the size range of ~200–300 nm (Figure 2a). z5-800 shows a much finer particle size of 30–50 nm (Figure 2b). However, z5-900 is apparently over-calcined (Figure 2c), and the particle size increases to ~200–400 nm with a severe agglomeration. The similar situation is presented in z5-950 but the agglomeration is much more severe (Figure 2d). Compared with the orthorhombic particles, the hexagonal ones are much finer and less agglomerated. This may be because the mismatch in radius induces some strain and defects in the lattice, impeding the particle growth [18]. Also, the high valence Zr^4+^ can suppress oxygen vacancy formation, restraining oxygen ion motion [19]. It is anticipated from the XRD and SEM results that the p-800 sample would have good magnetic properties while the z-800 one would exhibit good optical properties.

**Figure 3:**
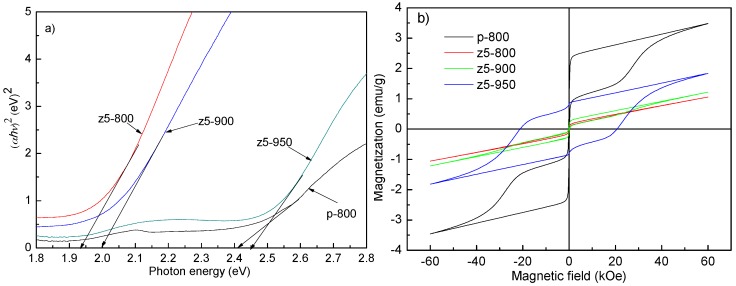
(**a**) Optical band gaps and (**b**) room-temperature magnetic hysteresis loops for p-800, z5-800, z5-800, and z5-950 samples.

YFeO_3_ is a semiconductor with a small optical band gap and has been adopted as a photocatalyst for compound decomposition. The optical properties are evaluated by optical band gaps obtained from the UV–visible absorption spectra [7]. Figure 3a shows the optical band gaps of p-800, z5-800, z5-900, and z5-950 samples, being 2.41, 1.96, 2.01, and 2.45 eV, respectively. The optical band gap of YFeO_3_ diminishes obviously from the orthorhombic to hexagonal phase. The excellent UV–visible absorption property of the hexagonal YFeO_3_ are related to two aspects. Firstly, the hexagonal YFeO_3_ possesses a small band gap, leading to a higher optical absorption. The shifting in the position charge-transfer band is ascribed to the modification in FeO_6_ local environment caused by the transformation from the orthorhombic to hexagonal phase [20]. Secondly, owing to a reduced particle size, the hexagonal phase can also increase the specific surface area and decrease the travelling distance of photo carriers during their transfer from inside to outside of the particle, facilitating the trapping of photo carriers on the surface [15].

Figure 3b represents the magnetic hysteresis loops of YFeO_3_ and YFe_0.95_Zr_0.05_O_3_ nanopowders at room temperature. The maximum magnetization (*M*_m_), remnant magnetization (*M*_r_), and coercive field (*H*_c_) of p-800 powders are about 3.49 emu/g, 0.88 emu/g, and 160 Oe, respectively. The hysteresis loops for hexagonal z5-800 and z5-900 powders shows an almost linear loop, resembling a paramagnetism. This is obviously ascribed to their high-purity hexagonal phase. For the z5-950 sample, apparent ferromagnetism in the antiferromagnetic YFeO_3_ material can be viewed but it is worse than that for the p-800 sample due to its large particle size and severe agglomeration. The optical and magnetic properties of the samples are consistent with their crystal structures and morphologies, i.e., the hexagonal YFeO_3_ possesses the better optical properties and the orthorhombic one exhibits the better magnetic properties, and the finer the particles, the better the corresponding properties are.

## 4. Conclusions

An easy and effective method of Zr doping is developed to synthesize YFeO_3_ nanoparticles with different phase compositions. For the undoped YFeO_3_, the pure orthorhombic phase can only be obtained and the calcination temperature must be 800 °C or over. The pure hexagonal YFeO_3_ can be synthesized between 800 and 900 °C with the aid of Zr doping. The hexagonal Zr-doped YFeO_3_ shows a narrower optical band gap (1.96 eV), while the orthorhombic YFeO_3_ owns better magnetic properties, being *M*_m_ = 3.49 emu/g and *H*_c_ = 160 Oe. The temperature range for the synthesis of hexagonal YFeO_3_ can be increased by ~200 °C due to Zr doping, making it easier to obtain a pure hexagonal YFeO_3_.
